# Cattle Meat and Offal Imported from the United States of America, Canada and Ireland to Japan (Prions)

**DOI:** 10.14252/foodsafetyfscj.D-20-00019

**Published:** 2020-09-25

**Authors:** Food Safety Commission of Japan

## Abstract

Food Safety Commission of Japan (FSCJ) was requested by the Ministry of Health, Labour and Welfare (MHLW) to conduct a risk assessment of cattle meat and offal imported from the United States of America (U.S.A.), Canada and Ireland. FSCJ assessed potential influences on bovine spongiform encephalopathy (BSE) risks to human health in cases of the alteration of cattle age to be allowed to import of cattle meat and offal from the three countries, from the current under 30 months of age to no age limitation, in line with the international standards for mitigating BSE risks. FSCJ judges that the control measures regarding “risks related to slaughtering and meat processing” are appropriately implemented in the three countries. FSCJ concludes that potential variations of BSE risks to human health by removing the age limit on cattle meat and offal excluding specified risk material (SRMs) imported from the three countries in line with the international standards is negligible.

## Conclusion in Brief

Food Safety Commission of Japan (FSCJ) was requested by the Ministry of Health, Labour and Welfare (MHLW) to conduct a risk assessment of cattle meat and offal imported from the United States of America (U.S.A.), Canada and Ireland. Using reference materials and documents submitted by the MHLW regarding the bovine spongiform encephalopathy (BSE) situations in the three countries, FSCJ assessed the risk of BSE agent in cattle meat and offal in relation to border measures such as slaughter age limit^1^^)^ of imports.

FSCJ assessed potential influences on BSE risks to human health in cases of the alteration of cattle age to be allowed to import of cattle meat and offal from the three countries, from the current under 30 months of age to no age limitation, in line with the international standards for mitigating BSE risks.

The cases of classical BSE continue to decrease and are scarcely reported in recent years worldwide.

Due to the decreased numbers of cattle exposed to BSE prion, the historical and existing risk factors are expectedly reduced to large extents in the three countries currently evaluated.

Therefore, even in countries where typical BSE has occurred in cattle born within the last 11 years, the risk of developing typical BSE is estimated to be extremely low as long as the control measures related to the historical and existing risk factors of BSE in live cattle are properly implemented. In addition, assuming the control measures are maintained at the same level as the current level, the frequency of occurrence is estimated to remain below the current level.

Under these situations, FSCJ decided to assess the risk of meat and offal through the verification of control measures such as specified risk material (SRMs) removal and ante-mortem inspection in cattle at the slaughter house of these countries. While an international standard [The OIE *Terrestrial Animal Health Code* (The *Terrestrial Code*) ] does not set a an age limit for trade in beef, FSCJ decided to evaluate whether or not the risk of variant Creutzfeldt-Jacob disease (vCJD) associated with intake of the BSE prion has reached to an extremely low level.

The results of the risk assessment are summarized below.

In the three countries, indigenous classical BSE cases have not been confirmed in the U.S.A., and few cases of indigenous classical BSE are confirmed in Canada and Ireland at present. Thus, the control measures against historical and existing risk factors (the *Terrestrial Code*)^2^^)^ are recognized to be effective toward preventing the incidence of classical BSE in these countries. Therefore, FSCJ judges the incidence of classical BSE continually to be quite unlikely and/or to remain below the current level.

According to the data on the body distribution of abnormal prion protein (PrP^Sc^) in the classical BSE-infected cattle, the amount distributed in the tissues other than SRMs is extremely low. Epidemiological information on vCJD cases supported this observation. Therefore, it is assumed that the removal of SRMs ensures, if any, the negligible intakes of PrP^Sc^ through meat and offal. Moreover, the ante-mortem inspections remove the cattle manifesting the clinical signs.

FSCJ judges that the control measures regarding “risks related to slaughtering and meat processing” are appropriately implemented in the three countries.

Furthermore, taking into account the interspecies barrier between human and bovine, FSCJ considers that vCJD is highly unlikely to develop in association with consumption of classical BSE prions through cattle meat and offal (excluding SRMs) imported from the three countries after the age limit is removed. Assuming that current risk control measures are continuously implemented as mentioned above. For the atypical BSE, the previous assessment on “BSE counter measures applied to domestic cattle” has concluded that vCJD is highly unlikely to develop in association with consumption of classical BSE prions through cattle meat and offal (excluding SRMs) under the continuous implementation of current control measures against BSE, and new findings affecting this conclusion are not available.

Considering thoroughly available evidence, FSCJ reached the following conclusion on the risk of BSE agent in cattle meat and offal imported from the U.S.A., Canada and Ireland by increasing the age limit from the current 30 months of age in line with the international standards. FSCJ concludes that potential variations of BSE risks to human health by removing the age limit on cattle meat and offal (excluding SRMs) imported from the three countries in line with the international standards is negligible.

FSCJ drew this conclusion of the assessment assuming that current risk control measures are continuously implemented. Therefore, risk management organizations should continuously collect related information, particularly regarding feed regulation, surveillance, ante-mortem inspection of slaughter and control on SRM removal.
